# Ionotropic and Metabotropic Proton-Sensing Receptors Involved in Airway Inflammation in Allergic Asthma

**DOI:** 10.1155/2014/712962

**Published:** 2014-08-13

**Authors:** Haruka Aoki, Chihiro Mogi, Fumikazu Okajima

**Affiliations:** Laboratory of Signal Transduction, Institute for Molecular and Cellular Regulation, Gunma University, Maebashi 371-8512, Japan

## Abstract

An acidic microenvironment has been shown to evoke a variety of airway responses, including cough, bronchoconstriction, airway hyperresponsiveness (AHR), infiltration of inflammatory cells in the lung, and stimulation of mucus hyperproduction. Except for the participation of transient receptor potential vanilloid-1 (TRPV1) and acid-sensing ion channels (ASICs) in severe acidic pH (of less than 6.0)-induced cough and bronchoconstriction through sensory neurons, the molecular mechanisms underlying extracellular acidic pH-induced actions in the airways have not been fully understood. Recent studies have revealed that ovarian cancer G protein-coupled receptor 1 (OGR1)-family G protein-coupled receptors, which sense pH of more than 6.0, are expressed in structural cells, such as airway smooth muscle cells and epithelial cells, and in inflammatory and immune cells, such as eosinophils and dendritic cells. They function in a variety of airway responses related to the pathophysiology of inflammatory diseases, including allergic asthma. In the present review, we discuss the roles of ionotropic TRPV1 and ASICs and metabotropic OGR1-family G protein-coupled receptors in the airway inflammation and AHR in asthma and respiratory diseases.

## 1. Introduction

Airway acidification has been shown to be attained by either the exogenous way, that is, the microaspiration of acid contents into the airway during gastroesophageal reflux and inhalation of low pH pollutant aerosol, or the endogenous way, that is, ischemia and inflammation of the airways in inflammatory diseases, such as asthma, cystic fibrosis, and chronic obstructive pulmonary disease (COPD) [1–3]. In ischemic and inflammatory situations, the stimulation of anaerobic glycolysis causes lactate production. In patients with asthma, it has been reported that airway pH reaches 5.2 to 7.1, depending on the severity: pH is normalized with corticosteroid therapy [1]. Although alteration of airway pH may serve an innate host defense capacity, that is, inhibiting the survival of bacteria in an acidic environment, it is also implicated in the pathophysiology of obstructive airway diseases. Thus, exposure to acids evokes a cough, bronchoconstriction, airway hyperreactivity (AHR), and microvascular leakage and stimulates mucus production [2]. However, molecular mechanisms underlying the extracellular acidic pH-induced actions in the airways have not been fully understood. In the present review, we discuss the proton-sensing mechanisms, focusing on proton-sensing ionotropic receptors, such as transient receptor potential vanilloid-1 (TRPV1) and acid-sensing ion channels (ASICs), and metabotropic ovarian cancer G protein-coupled receptor 1 (OGR1)-family G protein-coupled receptors (GPCRs), in the airway inflammation and AHR in asthma and respiratory diseases.

## 2. General Information Regarding Proton-Sensing Channels and OGR1-Family GPCRs

**Figure 1:**
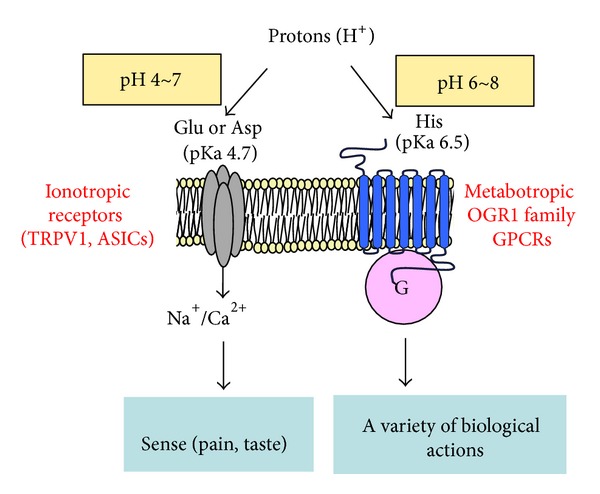
Ionotropic and metabotropic proton-sensing receptors. Extracellular acidification evokes a variety of airway responses. Ionotropic TRPV1 channel and ASICs mainly mediate severe acidic pH-induced cough, pain, and bronchoconstriction through sensory neurons, while OGR1-family GPCRs sense mild alkaline and mild acidic pH and exert a wide range of cellular actions in many types of structural and inflammatory cells.

The mammalian transient receptor potential (TRP) superfamily of nonselective cation channels encompasses 28 isotypes and is divided into six subfamilies, that is, TRPC, TRPV, TRPM, TRPA, TRPP, and TRPML. These channels are expressed in neurons and a wide range of cell types in many biological systems [4–6]. TRP channels have six putative transmembrane domains and a pore-forming loop between the fifth and sixth segments. They are thought to be composed of homo- or heterotetramers [5]. Among them, capsaicin-sensitive TRPV1 is activated by a diverse range of chemical and noxious stimuli, including protons [5–7]. TRPV1 senses relatively strong acidic pH of 4 to 5 through glutamic acid in the extracellular domain of the channel [7] (Figure 1). TRP channels other than TRPV1, including TRPA1, TRPV4, and TRPM8, are expressed in the respiratory system and involved in the regulation of airway functions [8–10]; however, whether protons practically trigger their channel activation remains unknown.

Another important family of proton-sensing channel is ASIC. ASICs are proposed to assemble as tetramers with homomeric or heteromeric subunits; each subunit consists of two transmembrane domains [11] (Figure 1). Six ASIC subunit proteins, encoded by four genes, have been identified: ASIC1a, ASIC1b, ASIC2a, ASIC2b, ASIC3, and ASIC4. ASICs are voltage-independent channels that mainly conduct Na^+^ [12]. Recent studies have demonstrated that ASICs activated by acidic pH play an important role in a wide range of physiological and pathophysiological processes such as nociception, mechanosensation, synaptic plasticity, and acidosis-mediated neuronal injury [11]. Histidine, glutamic acid, and aspartic acid may determine a broad range of optical pH of 4 to 7 for activation, depending on the subtypes [4, 13, 14]. Some forms of ASIC mRNAs have been detected in pulmonary sensory neurons [6].

In addition to TRPV1 and ASICs, there is increasing evidence that further acid-sensitive ion channels are involved in monitoring acidosis. These include TRP family ion channels other than TRPV1, including TRPV4, TRPC4, TRPC5, and TRPP2 (PKD2L1); two-pore domain K^+^ (K_2p_) channels; ionotropic purinoceptors (P2X); inward rectifier K^+^ channels; voltage-activated K^+^ channels; L-type Ca^2+^ channels; hyperpolarization-activated cyclic nucleotide gated channels; gap junction channels; and Cl^−^ channels [4]. Although most of these channels seem to be expressed and play important roles in the respiratory system, their extracellular proton-sensitivity has not, to our knowledge, been demonstrated for specific airway responses [10, 15–17].

**Figure 2:**
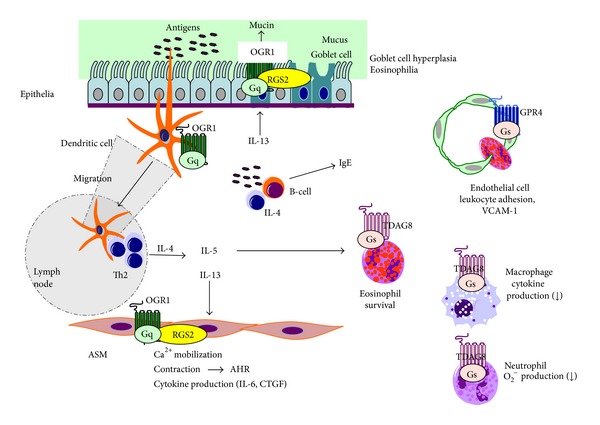
Role of OGR1-family GPCRs in Th2 polarization and subsequent airway inflammation and AHR. See the text in detail.

Recent studies suggest that OGR1-family GPCRs, including OGR1 (GPR68), GPR4, and T cell-death associated gene 8 (TDAG8 or GPR65), also sense extracellular protons and, thereby, stimulates a variety of cellular activities through several types of G proteins [18–21] (Figure 1). This receptor family is expressed not only in neurons but also in nonneuronal cells. OGR1-family GPCRs were previously described as GPCRs for lysolipids, such as sphingosylphosphorylcholine [19–21]. Ludwig et al. [18], however, first discovered that OGR1 and GPR4 sense extracellular protons and are coupled to G_q_ and G_s_, leading to activation of the phospholipase C/Ca^2+^ signaling pathway and the adenylyl cyclase/cAMP signaling pathway, respectively [18]. Later it was found that TDAG8 similarly senses extracellular protons, leading to the activation of the cAMP signaling pathway [22, 23]. OGR1-family GPCRs sense weak acidic to weak alkaline pH of 6 to 8 through histidine residues [18, 19, 24, 25]. G2A is also classified in this receptor family and is expressed by a broad range of immunoregulatory cell types, including macrophages, dendritic cells, neutrophils, mast cells, and T and B cells, and it is suggested to play an important role in innate and adaptive immunity [26]. However, the proton sensitivity of the receptor is very small and its role as a proton sensor has been questioned [27]. In the present review, therefore, we do not focus further on G2A. OGR1-family GPCRs are expressed in many cell types localized in the airways, and the cases in which their roles are demonstrated are summarized in Figure 2.

## 3. Role of Proton-Sensing Channels and GPCRs in the Airways

### 3.1. Bronchoconstriction and AHR

#### 3.1.1. Sensory Neurons

The proton-sensing TRPV1 channel and/or ASICs in sensory nerves have first been proposed to be involved in acidic pH-induced airway responses [2, 28, 29]. It has been established that TRPV1 on capsaicin-sensitive primary sensory neurons plays an important role in nociception and transmission of pain as a sensor of noxious stimuli [2, 4, 30]. The activation of TRPV1 on the sensory neurons by irritant compounds, including capsaicin and citric acid, generates reflex responses that, in turn, stimulate the release of tachykinins from the terminals of the sensory nerves, causing cough and bronchoconstriction [2, 5, 29]. The possibility that the afferent signals and neuropeptide release are coupled at the same nerve endings has also been proposed [31]. The neurogenic role of TRPV1 was based mainly upon experiments with selective agonists and antagonists. For example, capsazepine, a TRPV1-selective antagonist, and SR48968, a selective NK2 receptor antagonist, blocked citric acid inhalation-induced bronchoconstriction in guinea pig airways* in vivo* [32]. The role of TRPV1 in AHR has recently been demonstrated by the finding that oral administration of TRPV1-specific antagonists significantly attenuated AHR in the asthma model of OVA-sensitized guinea pigs [33]. In addition to TRPV1, involvement of ASICs in acidic pH-induced AHR was suggested by using selective ASIC inhibitors in the guinea pig tracheal rings* ex vivo* [28]. These results suggest that bronchoconstriction and AHR under acidic environments are indirectly mediated by sensory neurons through proton-sensing channels, such as TRPV1 and ASICs [6, 30]. In fact, no report has shown that TRPV1 and ASICs are expressed and are directly functioning in ASM cells, although vascular smooth muscle cells seem to express TRPV1 [8] and ASICs [34].

#### 3.1.2. Airway Smooth Muscle (ASM) Cells

The previous studies, however, did not rule out the direct actions of acidic pH on ASM cells. Ichimonji et al. [35] showed that extracellular acidification stimulates mRNA expression and protein production of IL-6, a proinflammatory cytokine, in association with the phosphorylation of extracellular signal-regulated kinase (ERK) and p38MAPK, in human ASM cells. They also showed that extracellular acidification induced an increase in intracellular Ca^2+^ concentration ([Ca^2+^]_i_) [35], which was accompanied by ASM cell contraction [36]. Acidification also induced expression of connective tissue growth factor (CTGF), a critical factor involved in the formation of extracellular matrix proteins and, hence, airway remodeling. TGF-*β*-induced CTGF expression was also enhanced by acidic pH [37]. In ASM cells, OGR1 is expressed at by far the highest levels among proton-sensing GPCRs [35]. The knockdown of OGR1 and G_q_ with their specific small interfering RNAs and an inhibition of G_q_ with YM-254890 attenuated the acidification-induced actions [35, 37]. These results suggest that extracellular acidification stimulates Ca^2+^ mobilization, inflammatory cytokine IL-6 and CTGF production, and contraction through OGR1/G_q_ in human ASM cells (Figure 2).

The role of OGR1 in AHR is supported by recent findings with RGS2- [38] and RGS5-deficient mice [39]. RGS2 and RGS5 are GTPase-activating proteins that inactivate G_q_ stimulated by G_q_-coupled receptors, including OGR1. Xie et al. [38] showed that RGS2 deficiency caused spontaneous AHR in response to methacholine without any prior antigen sensitization/challenge. They also showed that the loss of RGS2 augmented Ca^2+^ mobilization and the contraction of ASM cells, increased ASM mass, and stimulated ASM cell growth via ERK and phosphatidylinositol 3-kinase (PI3K) pathways. These results are highly consistent with those of* in vitro* studies with human ASM cells [35, 36]. Interestingly, asthma patients display lower expression of RGS2 in epithelial cells and ASM cells in lung and circulating monocytes [38]. RGS5-deficient mice also showed spontaneous AHR, even without antigen sensitization in association with enhanced Ca^2+^ mobilization, ERK activation, and ASM cell contraction [39]. In both cases, however, appreciable infiltration of inflammatory cells in the lung and production of cytokines, such as IL-4 and IL-5, was not observed [38, 39]. These results suggest that T-cell activation is not always required for GPCR-mediated asthma pathophysiology.

### 3.2. Mucus Hypersecretion in Epithelial Cells

Mucus hypersecretion is a common pathological feature of inflammatory airway diseases, including asthma. Liu et al. [40] have recently shown that an acidic pH of 6.4 stimulates mucin5AC (MUC5AC) secretion in the human bronchial epithelial cell line (16HBE) through the OGR1/G_q_/phospholipase C pathway (Figure 2). Knockdown of OGR1 and G_q_ expression with small interfering RNAs inhibited acidification-induced increases in [Ca^2+^]_i_ and mucin production. Similarly, overexpression of RGS2 protein attenuated acidic pH-induced cellular responses, whereas knockdown of RGS2 slightly but significantly enhanced these responses. These results suggest that airway acidification induces mucin production through the OGR1 and G_q_, of which activation can be regulated by RGS2. The same group has also shown that TRPV1 is expressed and citric acid (pH 5.0)-induced mucin secretion is mediated by Ca^2+^ influx via TRPV1 in the same cells [41]. These results suggest that epithelial cells utilize different proton-sensing machineries depending on the acidity of microenvironments; OGR1-family GPCRs sense mild alkaline to acidic pH of 8 to 6, whereas TRPV1 senses more acidic pH of 6 to 4, as described above.

### 3.3. Leukocyte Adhesion to and Leakage through Endothelial Cells

GPR4 has been shown to be expressed in endothelial cells and to be activated by extracellular acidification, leading to accumulation of cAMP, which may be relevant to normal blood vessel formation [42]. Chen et al. have recently shown that activation of GPR4 by acidosis increases endothelial cell adhesion of monocytes in association with the expression of adhesion molecules, such as VCAM-1 and ICAM-1, through the cAMP/Epac pathway [43]. Since VCAM-1 on the endothelium has been shown to mediate eosinophil adhesion and transmigration [44], this suggests that GPR4 is involved in the perivascular accumulation of eosinophils in the lung with inflammatory asthma (Figure 2). However, it remains unknown whether GPR4 deficiency affects eosinophilia in the airways with asthma.

### 3.4. Airway Eosinophilia due to Enhanced Survival of Eosinophils

Kottyan et al. [45] have reported that acidic pH increased eosinophil viability due to an inhibition of apoptosis, which was associated with an increase in cAMP accumulation. The acidic pH-induced increase in cell viability was attenuated by the adenylyl cyclase inhibitor and, in contrast, the phosphodiesterase inhibitor, the cell-permeable cAMP analog, and forskolin mimicked the acidic pH effect. The acidification-induced increase in eosinophil viability and cAMP accumulation was completely lost in the cells isolated from the TDAG8 knockout mice. These results suggest that eosinophil viability is increased in acidic microenvironments through the TDAG8/cAMP pathway (Figure 2). However, the mechanism by which cAMP inhibits eosinophil apoptosis remains unknown [45].

### 3.5. Cytokine and O_2_^−^ Production in Macrophages and Neutrophils

In macrophages, TDAG8 and OGR1 are expressed. Extracellular acidification inhibited lipopolysaccharide (LPS)-induced TNF-*α* and IL-6 protein and mRNA production in mouse peritoneal macrophages [46]. The inhibitory action on cytokine production by acidic pH was significantly attenuated in macrophages from TDAG8-deficient mice but not in those from OGR1-deficient mice. Further characterization revealed that acidic pH inhibited proinflammatory cytokine production through the TDAG8/G_s_/cAMP/PKA signaling pathway in mouse macrophages. The expression of TDAG8 was increased by glucocorticoid in the macrophage, which was associated with the enhancement of acidification-induced inhibition of TNF-*α* production [47]. Thus, TDAG8 seems to be indirectly involved in glucocorticoid-induced anti-inflammatory actions.

Extracellular acidification has been shown to induce human neutrophil activation, inducing an increase in [Ca^2+^]_i_, a shape-changing response, upregulation of the expression of CD18, an inhibition of apoptosis, and enhancement of agonist-induced H_2_O_2_ production [48]. These acidic pH-induced responses are accompanied by the activation of Akt and ERK. On the other hand, acidic pH has also been shown to inhibit some neutrophil functions. Extracellular acidic pH inhibits migration [49] and O_2_^−^ production in human neutrophils [50]. Although intracellular acidification was supposed to, in part, explain the acidic pH-induced actions [48, 50], the precise proton-sensing mechanisms remained largely unknown. Murata et al. [51] have shown that acidic pH inhibited fMLP- and C5a-induced superoxide anion production. The acidic pH effect was mimicked by cAMP increasing agents and attenuated by a PKA inhibitor. Moreover, acidic pH increased cAMP accumulation. TDAG8 is coupled to cAMP signaling pathways and is abundantly expressed in neutrophils. These data suggest that TDAG8 may mediate extracellular acidification-induced inhibition of O_2_^−^ production through cAMP [51].

Thus, TDAG8 is coupled to anti-inflammatory cAMP signaling pathways in macrophages and neutrophils. Consistently with an anti-inflammatory role, TDAG8-deficient mice showed exacerbation of anti-type II collagen antibody-induced arthritis, in which macrophages and neutrophils have been shown to play a critical role [52]. However, it remains uncharacterized whether the anti-inflammatory actions of TDAG8 in macrophages and neutrophils are involved in the pathophysiology of airway inflammation of asthma.

### 3.6. T-Cell Priming and Polarization by Dendritic Cells

Geffner and his colleagues have shown that extracellular acidification (pH 6.5) stimulates internalization of antigens; upregulated the expression of cell surface proteins, including CD11c, MHC class II, CD40, and CD86, involved in antigen presentation; and promoted an efficient MHC class I-restricted presentation of antigen peptides in dendritic cells (DCs) of C57BL/6 mice [53]. Antigen-pulsed DCs under acidic pH showed an improved efficacy for inducing both specific CD8^+^ cytotoxic T lymphocytes and specific antibody responses* in vivo* [53]. They further characterized acidic pH effects using human DCs and found that the transient exposure of human DCs to pH of 6.5 markedly increases several costimulatory proteins and improves the T-cell priming ability of DCs, which was associated with a dramatic increase in p38MAK-dependent IL-12 production. DC maturation by acidic pH stimulated the production of IFN-*γ*, but not of IL-4, by antigen-specific CD4^+^ T cells. These results suggest that extracellular acidification may contribute to the initiation of adaptive immune responses by DCs, favoring the development of the Th1 phenotype in humans [54].

Basu and Srivastava suggested that TRPV1 is expressed in mouse DCs and involved in their maturation [55]. However, the role of TRPV1 is controversial. TRPV1 expression was not confirmed and either capsaicin or acidic pH failed to elicit a change in [Ca^2+^]_i_ or the membrane current in mouse DCs [56]. Recent studies have shown that TRPV1 is expressed in human DCs; however, in this case, the channel seems to inactivate rather than activate their maturation [57]. The role of ASICs has also been suggested [58]. ASIC1, ASIC2, and ASIC3 are expressed in mouse DCs, and selective inhibitors for ASICs, such as amiloride and nonsteroidal anti-inflammatory drugs (NSAIDs), inhibited acidic pH 6.5-induced expression of cell-surface molecules CD11c, MHC class II, CD80, and CD86.

We have recently examined the role of proton-sensing GPCRs in DC functions [59]. Mouse DCs express OGR1-family receptors, including OGR1; the functional expression of OGR1 was confirmed by the extracellular acidic pH- and OGR1-dependent increase in [Ca^2+^]_i_. OVA-sensitized DCs from OGR1-deficient mice showed the reduction in the expression of CCR7, a chemokine receptor for mature DCs, and the migration responses to CCL19 and CCL21, ligands for CCR7, as compared with those from wild-type mice [59]. Thus, OGR1 seems to be functioning in the migratory process of DCs to draining lymph nodes (see the next section).

## 4. Role of Proton-Sensing Channels and OGR1-Family GPCRs in Asthma Models

Although the contribution of TRPV1 to acidic pH-induced cough and bronchoconstriction is well demonstrated as discussed, it remains to be proven whether TRPV1-mediated neurogenic inflammation plays a central role in asthma and other respiratory diseases. The inarticulate conclusion is based on the lack of obvious effects by gene targeting experiments; TRPV1 deficiency did not attenuate or rather enhanced the airway inflammation and AHR as induced by LPS [60], antigens [61, 62], and cigarette smoke [63] in TRPV1 knockout mice. The anti-inflammatory role of TRPV1 might be, in part, explained by the release of anti-inflammatory somatostatin from the sensory nerve terminals in response to TRPV1 stimulation [60]. Regardless, the role of TRPV1 in the airway inflammation and AHR remains to be established in mice. However, uncertain results with mice may be partly explained by the strain difference: the previous studies [60–63] used C57BL/6. Rehman et al. [64] have recently shown that TRPV1 knockdown with siRNA attenuates IL-13- and antigen-induced asthmatic features including airway inflammation and AHR in Balb/c mice.

In addition to proton-sensing channels, proton-sensing GPCRs also play an important role in the pathophysiology of asthma. As described above, Kottyan et al. have shown that TDAG8 deficiency causes the stimulation of eosinophil apoptosis and, thereby, reduces airway eosinophilia in OVA- and* Aspergillus fumigatus*-sensitization models of mice* in vivo* [45]. They have also shown increased expression of TDAG8 in lungs from OVA-sensitized mice and in nasal brushing samples from pediatric asthma patients. Unfortunately, whether AHR and airway inflammation as cardinal features of asthma are modulated by TDAG8* in vivo *has not been examined; however, it is noted that IL-13 production, which plays a role in these processes, was not affected by TDAG8 deficiency [45].

The findings of the expression of proton-sensing OGR1 in DC, a critical cell for antigen recognition and its presentation to T cells, and the involvement of OGR1 in the DC migration process [59] suggest participation of OGR1 in the pathophysiology of allergic asthma. Indeed, OGR1-deficient mice are resistant to the cardinal features of asthma, including airway eosinophilia, AHR, and goblet cell metaplasia, in association with a remarkable inhibition of the production of Th2 cytokines, including IL-4, IL-5, and IL-13, and OVA-specific IgE in an OVA-induced asthma model [59]. Intratracheal transfer to wild-type mice of OVA-primed bone marrow-derived DCs from OGR1-deficient mice developed lower AHR and eosinophilia as compared with the transfer of those from wild-type mice, which was associated with lower migratory activity to the peribronchial lymph nodes in OGR1-deficient DCs than in wild-type DCs. These results suggest that stimulation of OGR1 on DCs is critical for the early processes, that is, migration to lymph nodes and initiation of Th2 polarization, and, thereby, induces eosinophilia, airway inflammation, and AHR [59]. Since OGR1 is expressed in structural cells, including ASM cells and epithelial cells, and functions in a variety of cell-specific responses, the reduction of AHR and goblet hyperplasia in OGR1-deficient mice may be partly attributed to the reduction of acidification-induced constriction of ASM cells [35, 36] and mucin production in epithelial cells [40].

Finally, it should be noted that acidic pH modulates DCs leading to Th1 polarization in humans as described [51], which contrasts with the role of OGR1 in Th2 polarization in mice. The reason for the difference in the fate of T cells, that is, Th1 or Th2, is currently unknown. DCs express proton-sensing GPCRs other than OGR1, and OGR1-family GPCRs are expressed in neutrophils and macrophages as well. Therefore, differences in experimental conditions, for example, species, stimulants, antigens, and pH, may modify the state of DC activation and DC-T cell interaction, making naïve T cells polarize to Th1, Th2, Th17, or other phenotypes. How change in the pH microenvironment modulates DC function and T cell polarization warrants further study.

## 5. Conclusions

Proton-sensing channels, such as TRPV1 and ASICs, and OGR1-family GPCRs are expressed in structural cells, including ASM cells and epithelial cells, and inflammatory and immune cells, including eosinophils and DCs, and play a variety of roles in airway responses, depending on the optimum pH of proton-sensing channels and GPCRs. Cough and bronchoconstriction are activated by severe acidic pH of 4 to 5 and are mainly mediated by pH-sensing channels through sensory neurons, although pH-sensing OGR1 on ASM cells may also be involved in the cell constriction. At mild alkaline or mild acidic pH of more than 6, however, OGR1-family GPCRs may be the main receptors involved in the regulation of airway responses under pathophysiological situations, such as allergic asthma. Thus, ionotropic and metabotropic proton-sensing receptors may be therapeutic targets for inflammatory and ischemic diseases, such as asthma, for which drugs that are more specific and have fewer side effects are still required.

## List of Abbreviations

AHR: Airway hyperresponsiveness
ASIC: Acid-sensing ion channel
ASM: Airway smooth muscle
[Ca^2+^]_i_: Intracellular Ca^2+^ concentration
DC: Dendritic cell
ERK: Extracellular signal-regulated kinase
GPCR: G protein-coupled receptor
G_q_: G_q_ protein
G_s_: G_s_ protein
LPS: Lipopolysaccharide
OGR1: Ovarian cancer G protein-coupled receptor 1
PI3K: Phosphatidylinositol 3-kinase
RGS: Regulator of G protein signaling
TDAG8: T cell-death associated gene 8
TNF-*α*: Tumor necrosis factor-*α*
TRPV1: Transient receptor potential vanilloid-1
PKA: Protein kinase A.
